# Next-Generation *o*-Nitrobenzyl Photolabile Groups for Light-Directed Chemistry and Microarray Synthesis**

**DOI:** 10.1002/anie.201502125

**Published:** 2015-06-03

**Authors:** Nicole Kretschy, Ann-Katrin Holik, Veronika Somoza, Klaus-Peter Stengele, Mark M Somoza

**Affiliations:** Institute of Inorganic Chemistry, Faculty of Chemistry, University of ViennaAlthanstrasse 14 (UZA II), 1090 Vienna (Austria); Department of Nutritional and Physiological Chemistry, Faculty of Chemistry, University of Vienna(Austria); Christian Doppler Laboratory for Bioactive Aroma Compounds, University of Vienna(Austria); Roche DiagnosticsPenzberg (Germany)

**Keywords:** microarrays, photochemistry, photolysis, protecting groups

## Abstract

Light as an external trigger is a valuable and easily controllable tool for directing chemical reactions with high spatial and temporal accuracy. Two *o*-nitrobenzyl derivatives, benzoyl- and thiophenyl-NPPOC, undergo photo-deprotection with significantly improved efficiency over that of the commonly used NPPOC group. The two- and twelvefold increase in photo-deprotection efficiency was proven using photolithograph synthesis of microarrays.

Photolabile groups are widely used in chemical synthesis to extend available blocking strategies in a further orthogonal direction,[1] for photopolymerization, cross-linking, and functionalization in polymer chemistry,[2] for 3D patterning and fabrication,[3] and for the creation of biologically inactivated (caged) molecules that can be activated by light after they have been introduced into cells.[4] The most commonly used photolabile groups are *o*-nitrobenzyl derivatives; these have proved to be highly versatile, and are used to protect a wide variety of functional groups.[5] In contrast to chemically cleavable protecting groups, photolabile groups permit high-resolution spatial control of reactions when optical imaging systems deliver the light. Spatial control has proven to be particularly useful for the combinatorial synthesis of biopolymer microarrays. This approach, adopted for the industrial-scale synthesis of microarrays, can produce arrays with >10^6^ unique sequences per square centimeter.[6]

Photolithographic synthesis was first applied to peptide microarrays[7] using amino acids with the nitroveratryloxycarbonyl (NVOC) N-terminal protecting group, and then to DNA microarrays using 5'-(α-methyl-2-nitropiperonyl)oxycarbonyl (MeNPOC)[8] and dimethoxybenzoincarbonate (DMBOC)[9] phosphoramidites, but the relatively low yield obtained with these groups limits their use to microarrays of short oligomers.[10] The development of the 2-(2-nitrophenyl)propoxycarbonyl (NPPOC) group, with essentially quantitative yield and significantly higher photolysis quantum yield permitted the manufacture of microarrays of long oligonucleotides.[11] High photolytic efficiency, the product of the absorption coefficient and the photolysis quantum yield (*εφ*), is important in most applications of photolabile groups, not just because of higher yield and increased experimental throughput, but because minimizing irradiation proportionately reduces the risk of photochemical side reactions. The efficiency of the NPPOC group has led to its widespread use, not only for the synthesis of genomic DNA microarrays, but also for the synthesis of aptamer,[12] gene assembly,[13] RNA,[14] and peptide microarrays,[15] and in carbohydrate chemistry,[16] cleavable linkers,[17] and caging.[18]

The photolysis quantum yield of NPPOC is relatively high (0.41 in MeOH), but the low absorptivity (ε_365nm/MeOH_≈230 m^−1^ cm^−1^) has led to both the search for derivatives with higher absorptivity[19] and the development of photosensitization techniques based on intra- and intermolecular energy transfer from a triplet sensitizer.[20] For the most part, however, these derivatives and sensitizers have not proven to be robust replacements for NPPOC in the synthesis of complex microarrays of long oligomers.

Here we evaluate two NPPOC derivatives with greatly improved photolytic efficiencies in the synthesis of microarrays. The two derivatives, benzoyl-2-(2-nitrophenyl)propoxycarbonyl (Bz-NPPOC) and thiophenyl-2-(2-nitrophenyl)propoxycarbonyl (SPh-NPPOC) (Scheme 1), used as the 5'-hydroxyl protecting groups on DNA phosphoramidites, were tested to determine whether they can be used as effective replacements for NPPOC. We will show that microarrays synthesized with these groups are equivalent to or better than those synthesized with NPPOC, yet require far less light for photolysis. In the case of photolithographic synthesis, the lower amount of required light is a major advantage due to the low numerical aperture (NA) of the optical systems. Low NA is needed to generate sufficiently large depth-of-focus and to reduce synthesis errors due to scattered light, but greatly reduces the amount of usable light that can be obtained from any given source.[21]

**Scheme 1:**
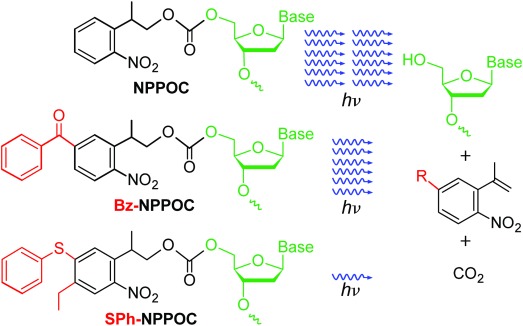
a) Structures and photocleavage products of DNA phosphoramidites with NPPOC, Bz-NPPOC, and SPh-NPPOC 5'-OH protecting groups.

The NPPOC, Bz-NPPOC, and SPh-NPPOC DNA phosphoramidites were evaluated using maskless array synthesis (MAS).[21, 22] MAS is a proven photolithographic approach for the in situ synthesis of high-density DNA microarrays for genomics applications, but it is now also used for the synthesis of arrays of RNA, peptides, and carbohydrates. MAS uses an array of digitally controlled micromirrors to direct light from a Hg lamp to the microarray synthesis surface. Light exposure is synchronized with chemical delivery in order to synthesize high-complexity microarrays (detailed methods given in the Supporting Information).

Figure 1 shows the absorption spectra of the thymidine phosphoramidites with NPPOC-, Bz-NPPOC, and SPh-NPPOC protecting groups, along with the spectral lines at *λ*=365, 405, and 436 nm from the Hg lamp. The absorbance from NPPOC and Bz-NPPOC are very similar in the relevant spectral region near *λ*=365 nm, so that the increased photolytic efficiency of Bz-NPPOC is due to increased quantum yield of photolysis. SPh-NPPOC absorption is seven times higher at *λ*=365 nm, and is significant until *λ*≈420 nm, but the spectral overlap with the line at *λ*=365 nm still accounts for >87 % of the total (Supporting Information). The spectral line at *λ*=436 nm is present, but does not contribute; the remaining Hg spectral lines are filtered out to prevent DNA damage and heating of the system.

**Figure 1:**
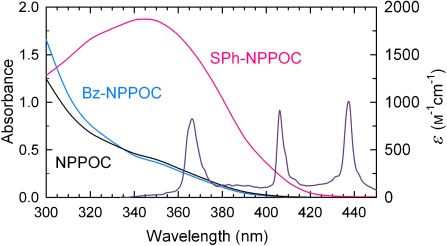
Absorption/extinction coefficient spectra of the phosphoramidites in DMSO, along with the spectral lines of the high-pressure Hg lamp at *λ*=365, 405, and 436 nm, as measured at the reaction site.

The light exposure necessary to remove the NPPOC, Bz-NPPOC, and SPh-NPPOC protecting groups was determined by creating microarrays with oligonucleotides sharing a common sequence but synthesized using a gradient of light exposures. These arrays were then hybridized with the fluorescently labeled complementary sequence and scanned. As the exposure increases, the sequence fidelity increases until the full hybridization signal is reached. Microarray synthesis using exposure gradients, followed by hybridization, is a highly sensitive test of photolysis efficiency since the value of each data point is determined by many consecutive photocleavage reactions, all of which need to be successful in order to generate a strong signal. Figure 2 shows the exposure gradients for the three photolabile groups used to synthesize mixed-base 25- and 60-mers. The radiant exposure values for Bz-NPPOC and SPh-NPPOC were scaled by 2.1 and 12.0, respectively, such that the data would overlap with the NPPOC data. The overlap of the curves indicates that the photolysis kinetics are equivalent but faster for Bz-NPPOC and far faster for SPh-NPPOC.

**Figure 2:**
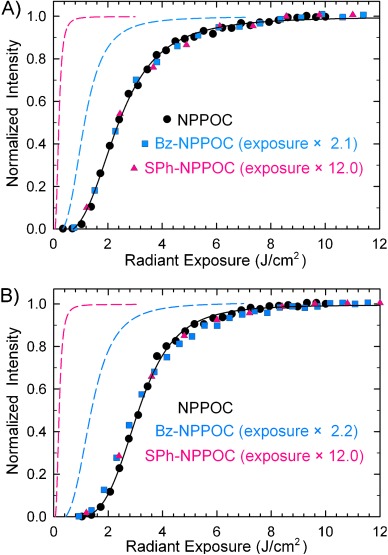
Hybridization intensities for A) 60-mer and B) 25-mer arrays synthesized with an exposure gradient using NPPOC (black circles), Bz-NPPOC (blue squares), and SPh-NPPOC (red triangles). Radiant exposure values for Bz-NPPOC and SPh-NPPOC are multiplied by 2.1 (2.2) and 12.0, respectively, with original data positions indicated by dashed lines.

Although the photolysis is very fast for Bz-NPPOC and SPh-NPPOC, the overall yield is also highly relevant. For an accurate comparison with NPPOC, a microarray was designed containing oligonucleotides that share a common sequence but were synthesized with two chemistries, NPPOC as a reference, and either Bz- or SPh-NPPOC.

The synthesis of the NPPOC-based oligomers used 6 J cm^−2^ exposures and the oligomers were compared, on the basis of hybridization intensity, with Bz-NPPOC and SPh-NPPOC oligomers synthesized using the proportionally lower exposures based on Figure 2, 2.7 and 0.5 J cm^−2^, respectively. Since NPPOC photolysis proceeds via a photoinduced β-elimination pathway that is favored by a small concentration of an amine base,[19b, 23] the rate of proton abstraction could be limiting under fast deprotection conditions. This might favor longer reactions performed with lower radiant power or the use of higher concentrations of the base in the exposure solvent (imidazole in DMSO). Relative to NPPOC, synthesis with Bz-NPPOC and SPh-NPPOC results in an equal or better hybridization signal under all tested conditions (Figure 3). Lower radiant power resulted in a modest improvement in hybridization intensity, but increased concentration of imidazole in the exposure solvent does not. A higher imidazole concentration does appear to improve signal homogeneity.

**Figure 3:**
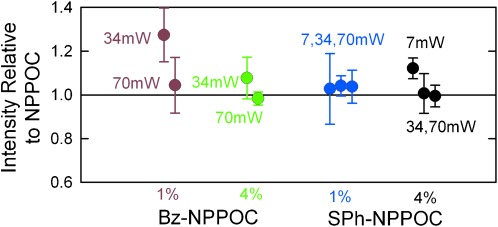
Hybridization intensity (relative to NPPOC) of Bz-NPPOC and SPh-NPPOC for several values of exposure radiant power (mW cm^−2^) and for photocleavage reactions carried out in either 1 % or 4 % imidazole in DMSO (w/v) as the exposure solvent. The error bars, the standard deviation of on-array replicates, serve as a measure of synthesis homogeneity.

The exposure gradients experiments, along with the measured extinction coefficients, provide accurate values for the relative quantum yield of photolysis. The absolute yields were determined by irradiating the 5'-OH-protected thymidine phosphoramidites in solution and quantifying the compounds and their photoproducts by HPLC. The photokinetic rate law for the concentration *c* of the starting compound is given by Equation (1).[24]


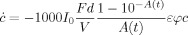
(1)

Here *I*_0_ is the irradiance, *F*, *d* and *V* are the exposure cross section, path length, and sample volume, respectively, and *A*(*t*) is the total absorbance of the sample. Figure 4 shows the decomposition kinetics and photokintetic factors, (1−10^−*A*(*t*)^)/*A*(*t*). The quantum yields *φ* were obtained by numerical integration of the photokintetic factor. Table 1 summarizes the results, which are highly consistent with the microarray data, with *εφ* for Bz-NPPOC double that of NPPOC. For SPh-NPPOC the photolysis efficiency is ten times greater that of NPPOC; this value is lower than the value (12 times greater) obtained in the array experiments due to the contribution of the 405 nm Hg line in array synthesis.

**Figure 4:**
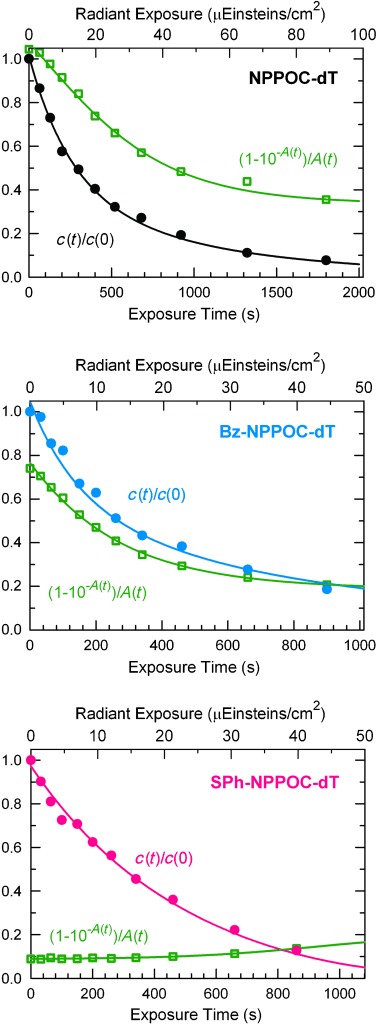
Kinetics of the photolysis of NPPOC-, Bz-NPPOC-, and SPh-NPPOC-protected thymidine with *λ*=365 nm light. Irradiance was 16.2 mW cm^−2^ for NPPOC and Bz-NPPOC, and 15.0 mW cm^−2^ for SPh-NPPOC. The *c*(*t*)/*c*(0) data were fitted according to Equation (1) and numerical integration of the photokintetic factor fit.

**Table 1:** Extinction, quantum yield, and photolytic efficiency for the photolabile groups^[a]^

Group	*ε*_365nm_ [m^−1^ cm^−1^]	*φ*_365nm_	*εφ*_365nm_ [m^−1^ cm^−1^]
NPPOC	260	0.40	104
Bz-NPPOC	240	0.84	202
SPh-NPPOC	1560	0.68	1064

[a] Molar absorptivity *ε*, photolysis quantum yield *φ*, photolytic efficiency *εφ*, all at *λ*=365 nm.

To test the potential of these groups under conditions representative of one of the most demanding applications of photolabile groups, high-density gene expression microarrays were synthesized using 5'-NPPOC-, Bz-NPPOC, and SPh-NPPOC phosphoramidites. The design included two replicates of each of at least three unique 60-mer probes for each of >45 000 human genes, as well as 20 to 100 replicates of quality-control and reference sequences, a total of 382 536 oligonucleotides. The arrays were tested by hybridization with labeled cDNA produced from a human colon adenocarcinoma cell line (Caco-2). Each of the three protecting-group approaches resulted in high-quality microarrays with similar quality assessment metrics results (Supporting Information). Figure 5 shows details of the images, along with the corresponding log_2_ scatter plots of control vs. treated samples of robust multiarray average (RMA) normalized data.[25]

**Figure 5:**
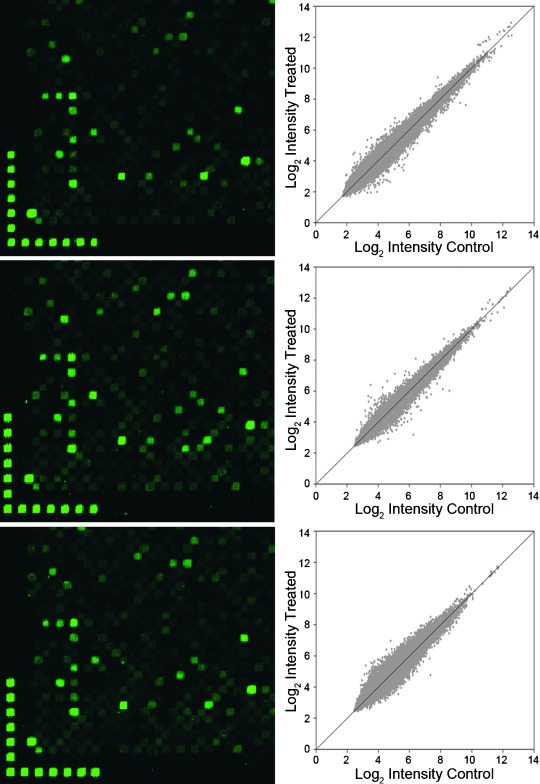
Left column: Details of 2.5 μm resolution scan images from gene expression microarrays synthesized with (top to bottom) NPPOC, Bz-NPPOC, and SPh-NPPOC and hybridized with Cy3-labeled cDNA. The size of each square is 14×14 μm. Right column: Scatterplots of the RMA-processed expression data from the gene expression microarrays synthesized with (top to bottom) NPPOC, Bz-NPPOC, and SPh-NPPOC.

In summary, two highly light-sensitive groups for light-triggered deprotection and spatio-selective synthesis, benzoyl-NPPOC and thiophenyl-NPPOC, have been shown to be superior replacements for NPPOC, one of the most commonly used photolabile groups in chemistry. The 2- and 12-fold increase in photodeprotection efficiency for Bz-NPPOC and SPh-NPPOC, respectively, significantly reduces the production time for photolithographic microarrays and these groups should prove to be useful replacements for NPPOC and other photolabile groups in many caging, synthetic, and triggering applications.

## Supporting Information

As a service to our authors and readers, this journal provides supporting information supplied by the authors. Such materials are peer reviewed and may be re-organized for online delivery, but are not copy-edited or typeset. Technical support issues arising from supporting information (other than missing files) should be addressed to the authors.

miscellaneous_information
