# Supplementary material for: Next-Generation o-Nitrobenzyl Photolabile Groups for Light-Directed Chemistry and Microarray Synthesis
Source: Angew Chem Int Ed Engl. 2015 Jun 3;54(29):8555–9. doi: 10.1002/anie.201502125 (PMC4531821; doi:10.1002/anie.201502125)
Supplement: Supplementary file 1 — miscellaneous_information [file anie0054-8555-sd1.pdf]

Angewandte Chemie
Eine Zeitschrift der Gesellschaft Deutscher Chemiker

Supporting Information

Next-Generation *o*-Nitrobenzyl Photolabile Groups for Light-Directed Chemistry and Microarray Synthesis**

*Nicole Kretschy, Ann-Katrin Holik, Veronika Somoza, Klaus-Peter Stengele, and Mark M. Somoza**

anie_201502125_sm_miscellaneous_information.pdf

Table of Contents

1. Microarray Synthesis Methods S2
    a. Microarray Substrate Preparation
    b. Microarray Synthesis
    c. Bz-NPPOC and SPh-NPPOC Phosphoramidites
    d. Microarray Synthesis Consumables
    e. Microarray Deprotection
    f. References

2. Exposure Gradient Experiments S5
    a. Microarray Design
    b. Hybridization

3. Gene Expression Microarrays S6
    a. Cell Culture
    b. Sample Preparation, Labeling and Data Extraction
    c. Gene Expression Microarray Hybridization Quality Control
    d. Gene Expression Microarray Treated vs. Control Log_2 Scatterplots
    e. Gene Expression Microarray Scan Images
    f. References

4. Absorption Spectra S9
    a. Methods
    b. Spectral overlap with Hg Lines
    c. Absorption Spectrum in Acetonitrile

5. Expedite Protocols and Coupling Sequences S11
    a. Light Exposure Gradient Microarrays
    b. Gene Expression Microarrays
    c. Synthesis Time of Gene Expression Microarrays
    d. References

6. HPLC Measurements of Quantum Yield S13
    a. Photochemical quantum yield determination
    b. Light exposure of samples
    c. HPLC determination of photoproduct concentration
    d. Data analysis and results
    e. References

1. Microarray Synthesis Methods

1.a Microarray Substrate Preparation

Schott Glass D microscope slides were functionalized with N-(3-triethoxysilylpropyl)-4-hydroxybutryamide (Gelest, Morrisville, PA). The slides were placed in stainless steel rack and gently agitated in a solution of 2% (v/v) of the silane and 0.1% acetic acid in 95:5 ethanol/water. After 4 hour at room temperature, the slides were rinsed twice for 15 min in the 95:5 aqueous ethanol and allowed to cure for overnight at 120°C under vacuum. After cooling to room temperature under vacuum, the slides are stored in a desiccator until use.

1.b Microarray Synthesis

The Maskless Array Synthesizer (MAS) instrument can be conceptually divided into two components, an optical system and a chemical delivery system. The chemical side consists of a Expedite 8909 nucleic acid synthesizer, which is used to deliver solvents and reagents to the functionalized glass surface where the microarray synthesis takes place. The optical system is similar to that of a photolithographic system, but it uses a digital micromirror device (Texas Instruments 0.7 XGA DMD) with 1024 x 768 individually addressable mirrors in place of photomasks to pattern the ultraviolet light from a mercury lamp.

The 350 W DC short arc mercury lamp (Newport 6286) is filtered using two 350-450 nm primary-range dichroic mirrors (Newport). The three unfiltered mercury lines (365, 405 and 436 nm) are further attenuated by multiple reflections in a reflective homogenizing light pipe, turning and imaging mirrors (Offner relay), and the DMD itself, before reaching the reaction cell where the photodeprotection takes place. The actual spectrum reaching the synthetic surface (**Figure 2**) was measured using a fiber spectrometer. The radient intensity of light reaching the reaction cell image plane was measured using a calibrated SÜSS Model 1000 UV intensity meter with a 365 nm probe (SÜSS MicroTec).

The pattern displayed on the DMD is imaged onto the synthesis surface, where array layout and oligonucleotide sequences are determined by selective removal of the photocleavable protecting groups. Reagent delivery and light exposures are synchronized and controlled by a computer, which also stores and orders the display of virtual masks on the DMD. The chemistry is similar to that used in conventional solid-phase synthesis. The primary modification is the use of phosphoramidites with a 5'-photocleavable protecting group. Upon absorption of a photon near 365 nm, and in the presence of a weak base, the NPPOC, Bz-NPPOC or SPh-NPPOC group drops off, leaving a hydroxyl

terminus which reacts with an activated phosphoramidite during the next synthesis cycle. Also, because there is no need to remove the DMT group as in conventional solid phase synthesis, there is no acid exposure and therefore no need for an oxidation step before deprotecting the 5‘-hydroxyl group. A final oxidation step is necessary to convert the DNA phosphites to phosphates before the microarray deprotection step. In the case of longer syntheses, intermittent oxidations steps were also used in addition to the final oxidation.

1.c Bz-NPPOC and SPh-NPPOC Phosphoramidites

The success of NPPOC derivatives lies in improving their absorptivities, their quantum yield for the deprotection reaction, or a combination of both of these. Much effort has been invested in exploring the substitutional space of the NPPOC phenyl ring in order to achieve improved photolytic efficiency (Bühler 2004), (Stengele 2005). Generally, substitutions *para* to the nitro, have led to the best results. Halogens in this position were associated with faster cleavage, presumably due to improved inter-system crossing resulting from a heavy-atom effect. A phenyl ring in this position also worked well (NBPPOC), presumably due to π-system extension. These results, together with the results on the photochemistry of ketoprofen (Laferriere 2004), suggested that a benzoyl group substitution in this position could also be effective. This led to the synthesis of Bz-NPPOC.

Photolysis improvement using triplet sensitization with thioxanthone (Wöll 2004) led to the synthesis of 5’-[3-nitrothioxanthone-2-(propyl-2-oxycarbonyl)]-nucleoside-3’-phosphoramidites (NTXPOC), which incorporates both benzoyl and thioxanthone structural elements onto NPPOC. Photolysis studies of NTXPOC indicated improved photolysis kintetics over NPPOC and Bz-NPPOC (Stengele 2005). SPh-NPPOC is closely related to NTXPOC, with the NTXPOC carbonyl group removed, following the indication that substitutions *para* to the nitro are primarily relevant to the photolysis. Removal of the carbonyl improved the quality of the photoprocess, most likely due to reduced damaging effect of radical initiation, which is well known for any thioxanthone moiety.

Details on the synthesis of the Bz-NPPOC and SPh-NPPOC phosphoramidites are given in (Bühler 2004), (Stengele 2004), and (Stengele 2013). The ethyl group on the SPh-NPPOC phenyl ring is a consequence of the synthetic route and is considered unlikely to affect the photolysis.

1.d Microarray Synthesis Materials

NPPOC phosphoramidites were purchased from Sigma-Aldrich (A112N01-01, C114N01-01, G114N01-01, T111N01-01) and diluted to a concentration of 30mM using Amidite Diluent (<30ppm water)

from Sigma-Aldrich (L010010-06). Bz-NPPOC and SPh-NPPOC were manufactured by NimbleGen Systems GmbH (Waldkraiburg, Germany) and diluted as above.

The phosphoramidites were activated using 0.25 M 4,5-dicyanoimidazole in acetonitrile (Biosolve Chimie). The exposure solvent consisted of 10 grams imidazole (Sigma-Aldrich 56750) per liter anhydrous DMSO (Biosolve Chimie). The oxidizer was tetrahyrofurane/water/pyridine/iodine 90.54/9.05/0.41/0.43 (v/v/v/w) (Sigma-Aldrich L860021). The synthesis washing steps used anhydrous acetonitrile. High purity Helium was used as blanket gas over the synthesis solvents and reagents, and also in the drying step of each cycle.

1.e Microarray Deprotection

After the synthesis, the nucleobase and phosphite protecting groups are removed by immersing the microarrays in 1:1 (v/v) ethylenediamine/ethanol for two hours at room temperature, then washed twice in deionized water and dried with argon.

1.f References

C. Agbavwe, C. Kim, D. Hong, K. Heinrich, T. Wang, M.M. Somoza, *Efficiency, error and yield in light-directed maskless synthesis of DNA microarrays*, Journal of Nanobiotechnology **2011**, 9.

S. Singh-Gasson, R. Green, Y. Yue, C. Nelson, F. Blattner, M. Sussman, F. Cerrina, *Maskless fabrication of light-directed oligonucleotide microarrays using a digital micromirror array*, Nature Biotechnology **1999**, 17, 974 - 978.

M. Sack, N. Kretschy, B. Rohm, V. Somoza, M.M. Somoza, *Simultaneous Light-Directed Synthesis of Mirror-Image Microarrays in a Photochemical Reaction Cell with Flare Suppression*, Analytical Chemistry **2013**, 85, 8513-8517.

K.-P. Stengele, J. Buehler, S. Buehler, E. Kvassiouk, R. Green, T. Prykota, W. Pfleiderer, *Recent Highlights on Photolithographic Oligonucleotide Array in Situ Synthesis*, Nucleosides, Nucleotides and Nucleic Acids **2005**, 24, 891-896.

S. Bühler, I. Lagoja, H. Giegrich, K.-P. Stengele, W. Pfleiderer, *New Types of Very Efficient Photolabile Protecting Groups Based upon the[2-(2-Nitrophenyl)propoxy]carbonyl (NPPOC) Moiety*, Helvetica Chimica Acta **2004**, 87, 620-659.

M. Laferriere, C.N. Sanrame, J.C. Scaiano, *A Remarkably Long-Lived Benzyl Carbanion*, Organic Letters **2004**, 6, 873-875.

D. Wöll, S. Walbert, K.-P. Stengele, T. J. Albert, T. Richmond, J. Norton, M. Singer, R. D. Green, W. Pfleiderer, U. E. Steiner, *Triplet-Sensitized Photodeprotection of Oligonucleotides in Solution and on Microarray Chips*, Helvetica Chimica Acta **2004**, 87, 28.

S. Bühler, M. Ott, W. Pfleiderer, *Photolabile protective groups for improved processes to prepare oligonucleotide arrays*, **2004**, WO 2004074300 A2.

K.-P. Stengele, *Diarylsulphid backbone containing photolabile protecting groups*, **2013**, WO 2012136604 A1.

2. Exposure Gradient Experiments

2.a Microarray Design

To measure the optimal light exposure parameters for removing the photocleavable protecting groups on the 5'-OH of the DNA phosphoramidites, microarrays were synthesized with a light-exposure gradient. For the duration of the exposure step of each synthesis cycle, 30 virtual masks were sent to the DMD in succession. The each of the masks was displayed for 1/30th of the time needed to reach the maximum exposure time required for each experiment. The first mask exposes all areas of the microarray where oligonucleotides are being synthesized. Each successive mask exposes fewer areas so that after the final mask is cleared from the DMD, specific areas on the surface contain oligonucleotides deprotected with the maximum exposure, while other areas contain oligonucleotides deprotected using successively lower exposure. The size of each exposed feature corresponded to a 5 x 5 array of DMD mirrors, or about 70 x 70 µM. 65 replicate features were synthesized at each of the 30 exposure levels and at random positions. In order to be able to make direct comparisons between NPPOC and Bz-NPPOC or SPh-NPPOC, the gradient microarrays also included 65 reference oligonucleotide features of the same sequence but synthesized with NPPOC and using a fixed radiant exposure of 6 J/cm^2. These features were synthesized immediately following the gradient synthesis by replacing the Bz-NPPOC or SPh-NPPOC phosphoramidites with NPPOC phosphoramidites in the synthesizer and resuming the synthesis. Control experiments, i.e., serial synthesis of the same oligonucleotide twice on the same microarray, show that the second gives a higher hybridization that the first. This is due, presumably, to chemical damage to the first during the synthesis of the second. The difference is modest, about 5%. For this reason, the intensity comparisons in Figure 3 likely slightly underestimate the intensity of Bz-NPPOC or SPh-NPPOC relative to NPPOC by about 5%. Nevertheless, it is important to compare synthesis chemistry and protocols on a single microarray because the comparison avoids the high inter-array variability introduced by hybridization and washing steps.

Exposure gradient experiments used the following sequences (5' to 3'):

60mer: GTT AAG CGA AGA AGA AAG TAG CGT GGC GCA CAG TTG CCC AAT CAA TTA CAC CCT CAT TTC

25mer: GTC ATC ATC ATG AAC CAC CCT GGT C

A 5 thymidine linker was synthesized before the hybridization sequences.

2.b Hybridization

The microarrays were incubated with the hybridization solution containing 150 µl 2x MES, 110 µl nuclease free water, 13.3 µl acetylated BSA and 26.7 µl of 100 nM 5'-Cy3-labeled complementary sequence using an adhesive hybridization chamber (Grace Biolabs SA200). During the incubation the microarray was rotated in an hybridization oven at 42 degrees. An air bubble of about a quarter of the chamber volume aids mixing during hybridization. After 4 hours for the exposure gradient experiments or 24 hours for the gene expression experiments, the chamber was removed in a petri dish filed with non-stringent wash buffer at 42°C. The microarrays were then washed in 50 ml centrifuge tubes containing 50 ml non-stringent wash buffer (SSPE; 0.9 M NaCl, 0.06 M phosphate, 6 mM EDTA, 0.01% Tween20) for 2 min., and then similarly washed with stringent wash buffer (100 mM MES, 0.1 M Na^+, 0.01% Tween20) for 1 min. Finally, the microarrays were dipped for a few seconds into final wash buffer (0.1× saline-sodium citrate buffer) and then dried with the use of a microarray centrifuge. Microarrays were scanned with an at a resolution of 2.5 µm. Data was extracted with NimbleScan 2.1 (Roche-NimbleGen).

3. Gene Expression Microarrays

3.a Cell culture

The human colon adenocarcinoma cell line Caco-2 was cultured in Dulbecco's modified Eagle medium supplemented with 10% fetal bovine serum, 4mM L-glutamine and 1% penicillin/streptomycin. Cells were maintained at 37°C and 5% CO_2 in a humidified incubator and passaged at 90% confluence. For microarray experiments, $4x10^5$ cells were seeded into 6-well plates and cultivated for 21 days to allow for enterocyte differentiation. Cultivation media was exchanged every second day.

3.b Sample Preparation, Labeling and Data Extraction

Fully differentiated Caco-2 cells were incubated with serum-free media or serum-free media containing 500 µM of a test substance. After 90 min, the cells were washed with ice-cold PBS and RNA isolated using the RNeasy Mini Kit (Qiagen, Hilden, Germany). The integrity of the isolated RNA was assessed by agarose gel electrophoresis in addition to photometrical analysis (Tecan, Menningen, Switzerland). A total of 10 µg RNA was reverse transcribed and simultaneously labeled using Cy3-labeled random nonamer primers (Tebu Bio, Offenbach, Germany) as described by Ouellet et al. The microarrays were hybridized under rotation at 42°C with a solution consisting of the Cy3-labeled cDNA, synthetic quality control oligonucleotides, acetylated BSA, herring sperm DNA and MES buffer. After 24 h the adhesive hybridization chamber was removed and the microarrays washed with a sequence of three buffers of increasing stringency: 2 min nonstringent wash buffer (SSPE; 900 mM NaCl, 60 mM phosphate, 6 mM EDTA, 0.01% Tween-20), 1 min stringent wash buffer (100 mM MES, 100 mM NacL, 0.01% Tween-20), 10 s final wash buffer (0.1 x SSC). The microarrays were dried with a microarray centrifuge and scanned at a resolution of 2.5 µm. The scanned images were analyzed using NimbleScan 2.1 (Roche-NimbleGen), employing robust multichip analysis (RMA) for normalization purposes. The extracted intensity data of each probe was $\log_2$ transformed and a scatter plot of control *vs.* treated samples created using SigmaPlot 11.0.

3.c Gene Expression Microarray Hybridization Quality Control

Three synthetic DNA oligomers, 5'-labeled with Cy3, were included in the hybridization buffer at concentrations of 3.7 nM. The sequences of these oligonucleotides are:

GAC CAG GGT GGT TCA TGA TGA TGA C,QC_25mer

GAT TTA GGT TTA CAA GTC TAC ACC GAA TTA ACA ACA AAA AAC ACG TTT TGG AG,ECOBioA1t_53mer

GAA ATG AGG GTG TAA TTG ATT GGG CAA CTG TGC GCC ACG CTA CTT TCT TCT TCG CTT AAC,ECOBioD2_60mer

The microarray layout was designed with 100 binding features for QC_25mer, 140 for ECOBioA1t_53mer, and 140 for EcoBioD2_60mer. The locations of each feature was randomized across the microarray. Several metrics were used to assess the hybridization quality of the synthetic oligonucleotides. **Table S1** contains the average intensity and coefficient of variation of the microarray features hybridized with each of the synthetic oligonucleotides. These values are extracted from the microarray intensity data prior to any image adjustments or normalization. Average intensity is calculated as the mean of the feature intensities for replicate features. This value can range from 0 to 65,535 and depends on, for example, the concentration of the labeled complementary oligonucleotide, the intensity of the scanner laser and the voltage of the

photomultiplier. The coefficient of variation is calculated as the standard deviation of the values divided by the mean.

Table S1. Average intensity and coefficient of variation values for labeled synthetic spike-in oligonucleotides used for quality control in the hybridization of the gene expression microarrays. Values are the average for two replicate gene expression microarrays of each kind. In each case, non-normalized probe intensity data is used.

	**NPPOC**		**Bz-NPPOC**		**SPh-NPPOC**	
	**Average Intensity**	**Coefficient of Variation**	**Average Intensity**	**Coefficient of Variation**	**Average Intensity**	**Coefficient of Variation**
**QC_25mer**	1270	0.25	3346	0.23	2356	0.25
**EcoBioA1_53mer**	986	0.27	2786	0.24	2494	0.25
**EcoBioD2_60mer**	1372	0.22	5016	0.22	2878	0.24

Table S2 contains quality metrics based on the microarray data processed using the Robust Multi-array Average (RMA) normalization. The particular implementation of RMA in NimbleScan screens for outlier probes and provides quantile normalization and background correction. Expression values are normalized values of the mean intensities for replicate features. Standard Error Expression values range from zero to one, where one indicates the highest confidence in the reliability of the measurement.

Table S2. Average expression and standard error values for labeled synthetic spike-in oligonucleotides used for quality control in the hybridization of the gene expression microarrays. Values are the average for two replicate gene expression microarrays of each kind. Results are from microarrays processed using Robust Multi-array Average (RMA) normalization.

	**NPPOC**		**Bz-NPPOC**		**SPh-NPPOC**	
	**Expression**	**Standard Error Expression**	**Expression**	**Standard Error Expression**	**Expression**	**Standard Error Expression**
**QC_25mer**	2609	0.92	2087	0.91	2127	0.96
**EcoBio1_60mer**	1860	0.92	1746	0.96	2241	0.98
**EcoBio2_60mer**	2834	0.92	3154	0.96	2703	0.98

3.d Gene Expression Microarray Treated vs. Control Log_2 Scatterplots

A common way to visualize microarray data is with intensity scatter plots using $\log_2$ transformed data. **Figure S1** shows scatterplots the RMA-processed expression data from the gene expression microarrays synthesized with NPPOC, Bz-NPPOC and SPh-NPPOC.

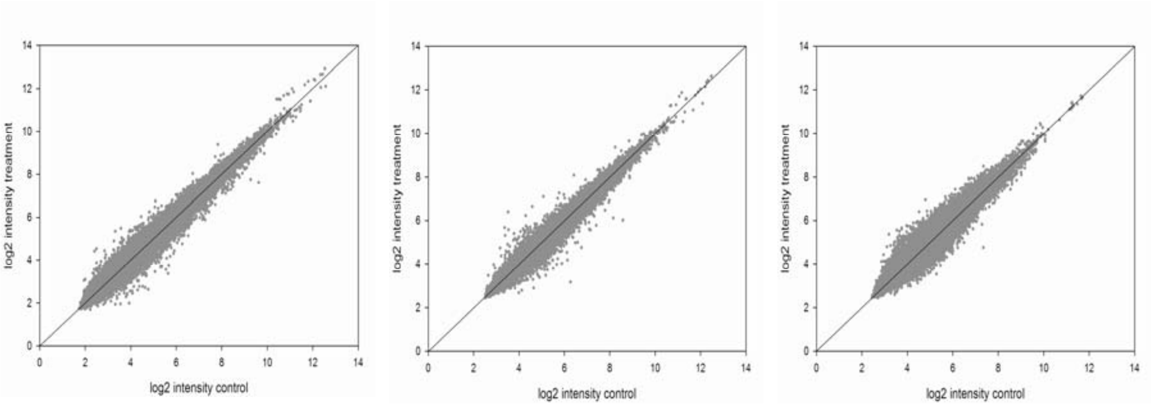

Figure S1*.* Scatterplots the RMA-processed expression data from the gene expression microarrays synthesized with NPPOC, Bz-NPPOC and SPh-NPPOC.

3.e Gene Expression Microarray Scan Images

Microarrays synthesized with NPPOC, Bz-NPPOC and SPh-NPPOC and then hybridized with labeled DNA, both synthetic and genomic, result in scanned images with very similar appearance. **Figure S2** shows a corner detail from microarray scans synthesized with the three chemistries.

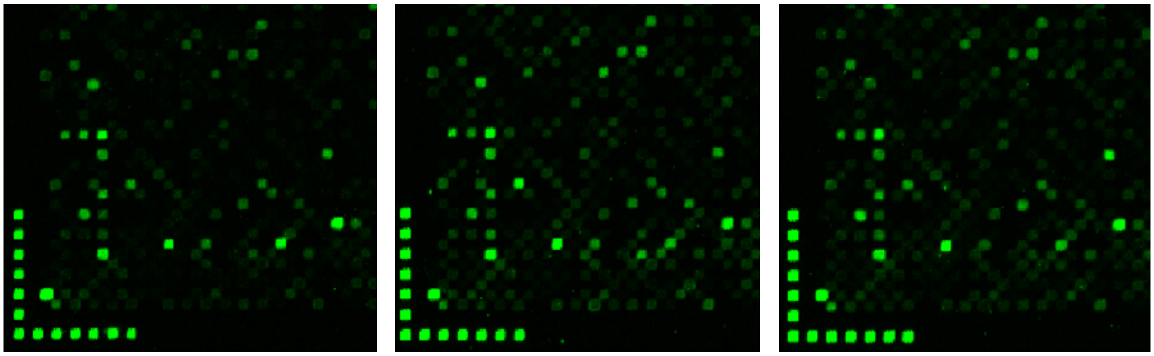

Figure S2. Detail of scan image from gene expression microarrays synthesized with (from left to right) NPPOC, Bz-NPPOC and SPh-NPPOC. The resolution of the scans is 2.5µm and the size of each square-shaped feature is ~14×14 µm.

3.f References

M. Ouellet, P.D. Adams, J.D. Keasling, A. Mukhopadhyay, *A rapid and inexpensive labeling method for microarray gene expression analysis,* BMC Biotechnology **2009**, 9, 97.

R. A. Irizarry, B. Hobbs, F. Collin, Y. D. Beazer-Barclay, K. J. Antonellis, U. Scherf, T. P. Speed *Exploration, normalization, and summaries of high density oligonucleotide array probe level data,* Biostatistics, **2003**, 4 (2), 249-264.

R. A. Irizarry, B. M. Bolstad, F. Collin, L. M. Cope, B. Hobbs, T. P. Speed, *Summaries of Affymetrix GeneChip probe level data,* Nucleic Acids Research, **2003**, 31 (4): e15.

4. Absorption Spectra

4.a Methods

Stock solutions of 30mM phosphoramidites were diluted with acetonitrile or DMSO to final concentrations of 1mM. Measurements were performed in a 1 cm path length cuvette using a Tecan Infinite M 1000 PRO.

4.b Spectral overlap with Hg Lines

To estimate the relative contribution to photo-deprotection of the mercury lines at 365, 405 and 436 nm, the absorption spectra of Bz-NPPOC and SPh-NPPOC in DMSO the spectral overlap functions, defined as the pointwise product of the absorption and emissions functions, were calculated. These functions are plotted (normalized to the peak of SPh-NPPOC) in **Figure S3**; the overlap function for NPPOC is essentially the same as that of Bz-NPPOC due to their very similar absorbance near 365nm. The plots show that for NPPOC and Bz-NPPOC, only the 365 nm mercury line contributes to photodeprotection, due to the low absorbance of both molecules at 405 and 436 nm. Even though only SPh-NPPOC overlaps significantly with the 405nm line, the overlap with the 365 nm line is far greater. The spectral overlap function of SPh-NPPOC in **Figure S3** is 7.8 times greater than that of Bz-NPPOC.

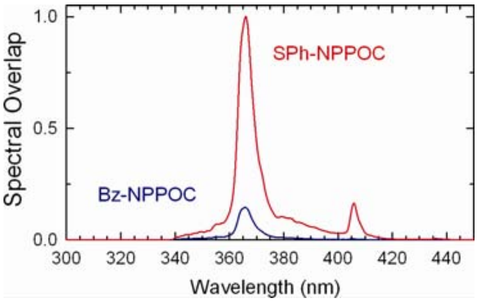

Figure S3. Overlap between the three mercury lines at 365, 405 and 436 nm (Figure 2) and the absorption spectra of Bz-NPPOC and SPh-NPPOC in DMSO.

4.c Absorption Spectrum in Acetonitrile

Although the exposure takes place with the photolabile groups in DMSO, reference absorption spectra were also obtained in acetonitrile. Acetonitrile, with small amounts of an amine base, can also be used as an exposure solvent. Such bases include imidazole, piperidine, DBU and DIPEA. The absorptions spectra in acetonitrile (**Figure S4**) are similar to those in DMSO, however, in DMSO the extinction coefficent is lower, but the shift of the spectra to the red largely compensates for this, resulting in similar extinction coefficients at 365nm.

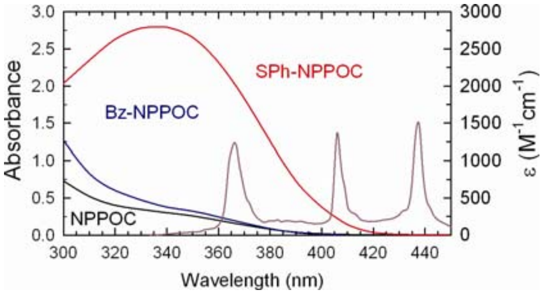

Figure S4. Absorption/extinction coefficient spectra of the phosphoramidites in acetonitrile, along with the high pressure Hg lines at 365, 405 and 436 nm, as measured at the reaction site.

5. Expedite protocols and coupling sequences

5.a Light exposure gradient microarrays

Coupling sequence 25mer (3’ to 5’):
sstttttCTGGTCCCACCAAGTACTACTACT7

Coupling sequence 60mer (3’ to 5’):
sstttttCTTTACTCCCACATTAACTAACCCGTTGACAC7CGGTGCGATGAAAGAAGAAGCGAA887

s = cleaning and priming cycle
t = dT coupling cycle for linker
A, C, G, T = coupling cycles without oxidation
7, 8 = dG, dT coupling cycles with oxidation

Table S3. Representative coupling protocol for exposure gradients. Protocol for dT, protocols for the other phosphoramidites are equivalent.

**$Coupling**		(pulses)	(sec)	
1 /*Wsh	*/ PULSE	20	0	"Flush system with Wsh"
2 /*Act	*/ PULSE	6	0	"Act"
21 /*T + Act	*/ PULSE	5	0	"Couple T"
2 /*Act	*/ PULSE	6	0	"Chase with Act"
1 /*Wsh	*/ PULSE	3	60	"Couple monomer"
1 /*Wsh	*/ PULSE	10	0	"Flush system with Wsh"
**$Capping**				
40 /*Gas A	*/ PULSE	1	30	"Dry column"
**$Oxidizing**				
*15/*Ox*	**/ PULSE*	*30*	*0*	*“Ox to column”*
*12 /*Wsh A*	**/ PULSE*	*20*	*0*	*“Flush system with Wsh A"*
17 /*Aux	*/ PULSE	15	0	"Exposure Solvent"
130 /*Event 2 Out	*/ NA	4	3	"Event 2 Out"
17 /*Aux	*/ PULSE	W	X	"Exposure Solvent"
12 /*Wsh A	*/ PULSE	Y	Z	"Flush system with Wsh A"
12 /*Wsh A	*/ PULSE	10	0	“Flush system with Wsh A"

The first two steps under the $Oxidizing header are omitted for non-oxidized cycles.

NPPOC: W = 40, X = 135 (exposure takes place during time X + Z)
Y = 10, Z = 15

Bz- & SPh-NPPOC: W = 35, X = 80
Y = 10, Z = 10

Table S4. Exposure parameters for gradient experiments. Values are for the longest exposure on each array.

	Time (s)	Radiant Power W/cm^2	Radiant Intensity (J/cm^2)
NPPOC	150	0.067	10
Bz-NPPOC	90	0.034	3
	90	0.070	6.3
SPh-NPPOC	90	0.07	0.63
	90	0.034	3
	90	0.070	6.3

5.b Gene Expression Microarrays

Coupling sequence (3' to 5'):

ssTTTTTACGTACGTACGTACGTACGTACGTACGTACGTACG8ACGTACGTACGTACGTACGTACGTAC
GTACGTACGTACG8ACGTACGTACGTACGTACGTACGTACGTACGTACGTACG8ACGTACGTACGTAC
GTACGTACGTACGTACG8ACGTACG8

s = cleaning and priming cycle
A, C, G, T = coupling cycles without oxidation
8 = dT coupling cycles with oxidation

Table S5. Representative coupling protocol for gene expression microarrays. Protocol for dT, protocols for the other phosphoramidites are equivalent.

**$Coupling**		(pulses)	(sec)	
1 /*Wsh	*/ PULSE	20	0	"Flush system with Wsh"
2 /*Act	*/ PULSE	6	0	"Act"
21 /*T + Act	*/ PULSE	5	0	"Couple T"
2 /*Act	*/ PULSE	6	0	"Chase with Act"
1 /*Wsh	*/ PULSE	3	50	"Couple monomer"
1 /*Wsh	*/ PULSE	10	0	"Flush system with Wsh"
**$Capping**				
40 /*Gas A	*/ PULSE	1	30	"Dry column"
**$Oxidizing**				
*15/*Ox*	**/ PULSE*	*30*	*0*	*"Ox to column"*
*12 /*Wsh A*	**/ PULSE*	*20*	*0*	*"Flush system with Wsh A"*
17 /*Aux	*/ PULSE	15	0	"Exposure Solvent"
130 /*Event 2 Out	*/ NA	4	3	"Event 2 Out"
17 /*Aux	*/ PULSE	W	X	"Exposure Solvent"
12 /*Wsh A	*/ PULSE	Y	Z	"Flush system with Wsh A"
12 /*Wsh A	*/ PULSE	20	0	"Flush system with Wsh A"

The first two steps under the $Oxidizing header are omitted for non-oxidized cycles.

NPPOC: W = 10, X = 54 (exposure takes place during time X + Z)
Y = 5, Z = 10

Bz-NPPOC: W = 10, X = 27
Y = 2, Z = 5

SPh-NPPOC: W = 0, X = 0 (steps omitted since exposure solvent train is long enough to fill the cell for the next 7 pulses below)
Y = 7, Z = 14

5.c Synthesis Time of Gene Expression Microarrays

The primary advantages of the Bz- NPPOC and SPh-NPPOC groups is the 2-fold and 12-fold decrease in exposure necessary for deprotection in comparision with NPPOC. In microarray synthesis

applications, other chemistry is also required, such as the phosphoramidite coupling reaction, as well as washing steps, as shown in **Section 5.b**. In our synthesis protocol (**Table S5**), the total synthesis times for gene expression microarrays (60mer probes on dT 5-mer linkers; 160 synthesis cycles) was 8 hrs 7 min for NPPOC (radiant exposure of 70mw/cm^2), 6 hrs 42 min for Bz-NPPOC (radiant exposure of 70mw/cm^2), and 5 hrs 50 min for SPh-NPPOC (radiant exposure of 70mw/cm^2). Since two mirror-image arrays are synthesized at once (Sack 2013), the average time per individual array is half of the time mentioned above. In addition, there is a set-up time of about 15 minutes in all cases. Since the exposure time can now be greatly reduced with the use of SPh-NPPOC, we decided to experiment with the other time parameters in order to further reduce the overall synthesis time. These results will be published separately (Sack 2015), but resulted in a synthesis time of ~3 hrs (70 seconds/cycle) for a pair of gene expression microarrays of the same design as above. Short synthesis times primarily increases synthesis throughput and decreases cost. With a synthesis time exceeding 8 hours, it is straighforward to perform one synthesis per day, and possible to perform two. However with a synthesis time of ~3 hours, it is straighforward to start three syntheses in a 8 hour normal work day and possible to perform 7 syntheses in 24 hours in an industrial setting (per array synthesizer).

Reduced light requirement for microarray synthesis, and other applications of photolabile groups, will also facilitate or enable the use of solid state UV sources (UV LEDs and solid state UV lasers) that have been introduced in recent years, but which are not as bright as traditional mercury sources.

5.d References

M. Sack, N. Kretschy, B. Rohm, V. Somoza, M.M. Somoza, *Simultaneous Light-Directed Synthesis of Mirror-Image Microarrays in a Photochemical Reaction Cell with Flare Suppression*, Analytical Chemistry, **2013**, 85, 8513-8517.

M. Sack, A.-K. Holik, V. Somoza, M. M. Somoza, *Express Photolithographic DNA Microarray Synthesis with Optimized Chemistry and High-Efficiency Photolabile Groups*, **2015**, in preparation.

6. HPLC Measurements of Quantum Yield

6.a Photochemical quantum yield determination

The microarray exposure gradients experiments, in conjunction with measured extinction coefficients, provide very accurate values for the relative quantum yield of photolysis, but cannot be

used to independently determine the absolute quantum yields. While the photolysis quantum yield for NPPOC has been previously measured in methanol (Wöll 2006), we decided to measure the photolysis quantum yield of NPPOC, Bz-NPPOC and SPh-NPPOC in order to be certain of their values in DMSO.

The 5'-NPPOC, -Bz-NPPOC and -SPh-NPPOC-thymidine solutions in DMSO were separately exposed to irradiation at 365nm for defined periods of time. The products were separated by HPLC and analyzed by UV spectroscopy. The photolysis quantum yield φ can be determined by using the following equation (**S1**) to fit the observed decay kinetics of the starting compound (Mauser 1998):

(**Eqn. S1**)
$$\frac{dc(t)}{dt} = -1000 I_0 \frac{Fd}{V} \frac{1-10^{-A(t)}}{A(t)} \varepsilon \varphi c(t)$$

The rate of change of the concentration $c(t)$ of the compound of interest depends on the photon irradiance I_0, the exposure area F, the path length through the sample d, the total volume of the sample V, the total absorbance of the solution as a function of exposure $A(t)$, the molar extinction coefficient ε of the compound of interest, and the quantum yield for photolysis φ. The factor of 1000 allows for the use of customary units: moles per liter (M) for concentration and cm^2, cm, cm^3 and $M^{-1}\,cm^{-1}$ for F, d, V, and ε, respectively, and mol $cm^{-2}\,s^{-1}$ (E $cm^{-2}\,s^{-1}$) for I_0. The derivation of the rate equation assumes the starting compound and photo-byproducts are homogenously distributed in the solution during the exposure (Mauser 1998).

This differential equation can be solved by the method of separation of variables and numerical integration of the photokinetic factor: $(1-10^{-A(t)})/A(t)$ in the general case when the solution absorbance changes with irradiance:

(**Eqn. S2**)
$$\int_{c_0}^{c(t)} \frac{1}{c} dc = -1000 I_0 \frac{Fd}{V} \varepsilon \varphi \int_0^t \frac{1-10^{-A(t)}}{A(t)} dt$$

(**Eqn. S3**)
$$\frac{c(t)}{c(0)} = e^{-1000 I_0 \frac{Fd}{V} \varepsilon \varphi \int_0^t \frac{1-10^{-A(t)}}{A(t)} dt}$$

Since the anti-derivative of 1/c in **Eqn. S2** is the natural logarithm of c, **Eqn. S3** results from applying the exponential function to both sides of the equation. Only the value for quantum yield for photolysis φ is unknown. The values for $A(t)$ are obtained by measuring the absorbance of the exposed solutions after an aliquot has been separated for the HPLC measurements.

6.b Light exposure of samples

The 5′-NPPOC, -Bz-NPPOC and -SPh-NPPOC-thymidine solutions in exposure solvent (1% imidiazole in DMSO) were exposed in a standard 1 cm path length quartz cuvette. The cuvette was masked with black tape to define a rectangular 0.5 cm high by 1 cm wide transparent exposure window into the cuvette. The exposure window was elevated 4mm above the bottom of the cuvette. The bottom 4 mm of the cuvette were occupied with a magnetic stir bar. One milliliter of the appropriate solution was used in each experiment so that Fd/V = ½. A conventional benchtop magnetic stirrer, set at the maximum rate of rotation, was used to maintain the homogeneity of the solution during the exposures. The ability of the magnetic stirring to maintain homogeneity was evaluated by adding 1 µL drops of concentrated Cy3 dye in acetonitrile to 1 mL of rapidly stirring DMSO. The solution color was observed to become homogenous on a time scale of approximately one second or less. The irradiance for the experiments was chosen so that the estimated half-life of the starting compound would be greater than 100 times the mixing time, in order to maintain the homogeneity requirement assumed in the derivation of the differential **Eqn. S1**.

To measure the quantum yield at a defined wavelength of light, 365nm, a Schott UG1 filter was used to block the 405 and 436 nm lines from the mercury lamp. The intensity of 365 nm light reaching the cuvette window was measured using a calibrated SÜSS Model 1000 UV intensity meter with a 365 nm probe (SÜSS MicroTec) covered with the UG1 filter to account for the ~25% attenuation of transmission through the filter.

The samples were degassed with helium and exposed under a helium blanket in a quartz sealed cuvette. Exposures were for 0, 64, 128, 200, 300, 400, 520, 680, 920, 1320 and 1800 seconds at 16.2mW/cm^2 (4.94 x 10^{-8} einsteins cm^{-2} s^{-1}) for NPPOC-dT; for 0, 32, 64, 100, 150, 200, 260, 340, 460, 660 and 900 seconds at 16.2mW/cm^2 (4.94 x 10^{-8} einsteins cm^{-2} s^{-1}) for Bz-NPPOC-dT; and for 0, 32, 64, 100, 150, 200, 260, 340, 460, 660 and 860 seconds at 15.0mW/cm^2 (4.57 x 10^{-8} einsteins cm^{-2} s^{-1}) for SPh-NPPOC-dT.

6.c HPLC determination of photoproduct concentration

The analysis was carried out using a Dionex Ultimate 3000 system (Thermo Fisher Scientific, Vienna, Austria) with a Dionex Ultimate 3000 RS Diode Array Detector set to a detection wavelength of 190 nm. A Phenomenex Luna 5u reverse phase column (250 x 3.00 mm, 5 micron, 100A, Phenomenex, Aschaffenburg, Germany) was employed. The column was equilibrated for 5 min after each run. The gradient was set to begin 2 min after the injection at initial conditions of 80% water and 20% acetonitrile (Carl Roth Rotisolv HPLC-grade) and reached 100% acetonitrile after 15 min. After 5 min of pure acetonitrile the gradient was set to return to initial conditions within 6 min. The NPPOC, Bz-

NPPOC and SPh-NPPOC samples were diluted ten-fold with acetonitrile acidified with 0.3% formic acid (Sigma-Aldrich) and passed through a 0.2 µm filter before analysis. For quantification purposes mixed standards of Bz-NPPOC, SPh-NPPOC and NPPOC were prepared giving the following calibration curves: 0.378x+0.747 (Bz-NPPOC), 0.250x-0.08 (SPh-NPPOC), 0.250x+0.602. **Figures S5**, **S6**, and **S7**, show the HPLC chromatograms for NPPOC, Bz-NPPOC, and SPh-NPPOC, respectively.

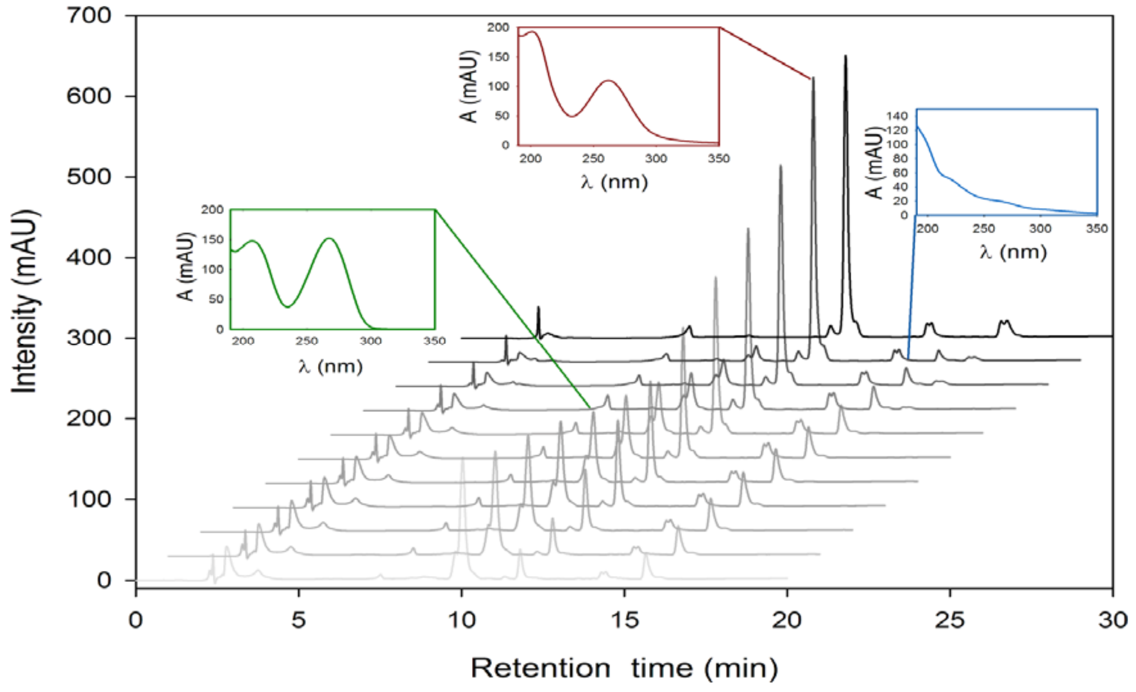

Figure S5. Overlay chromatogram of original NPPOC sample (back) and increasing exposure degree (front) showing retention time and UV trace of NPPOC (11.83 min) and exposure products thymidine (10.02 min) and styrene (15.73 min).

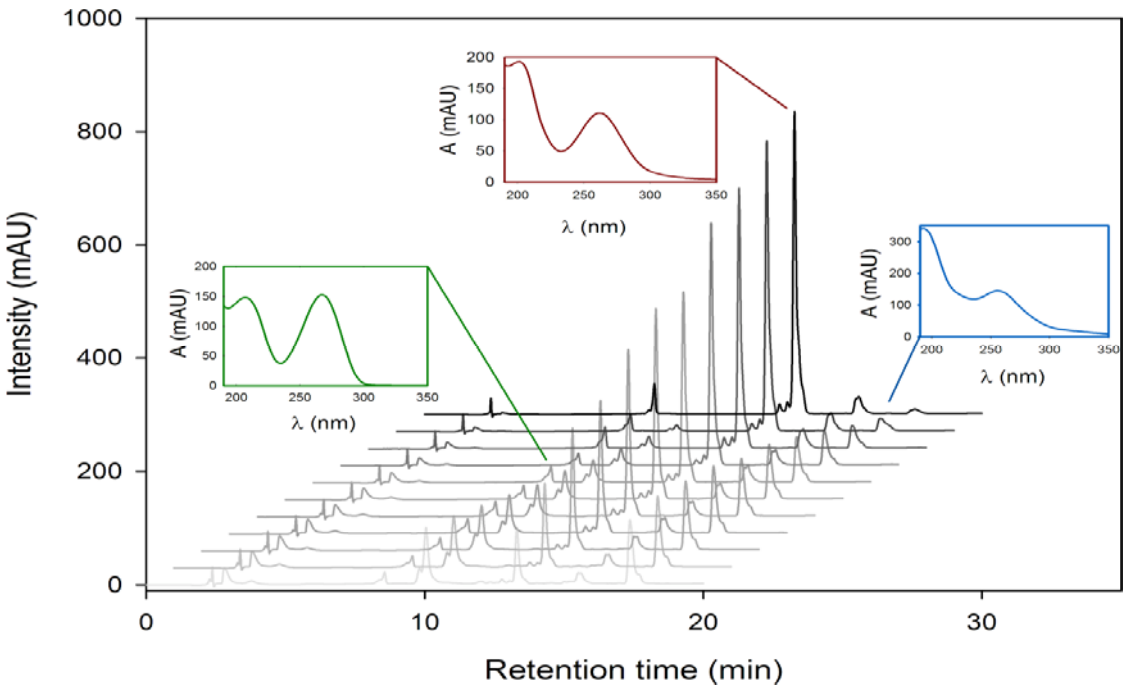

Figure S6. Overlay chromatogram of original Bz-NPPOC sample (back) and increasing exposure degree (front) showing retention time and UV trace of Bz-NPPOC (13.34 min) and exposure products thymidine (10.02 min) and Bz-styrene (17.40 min).

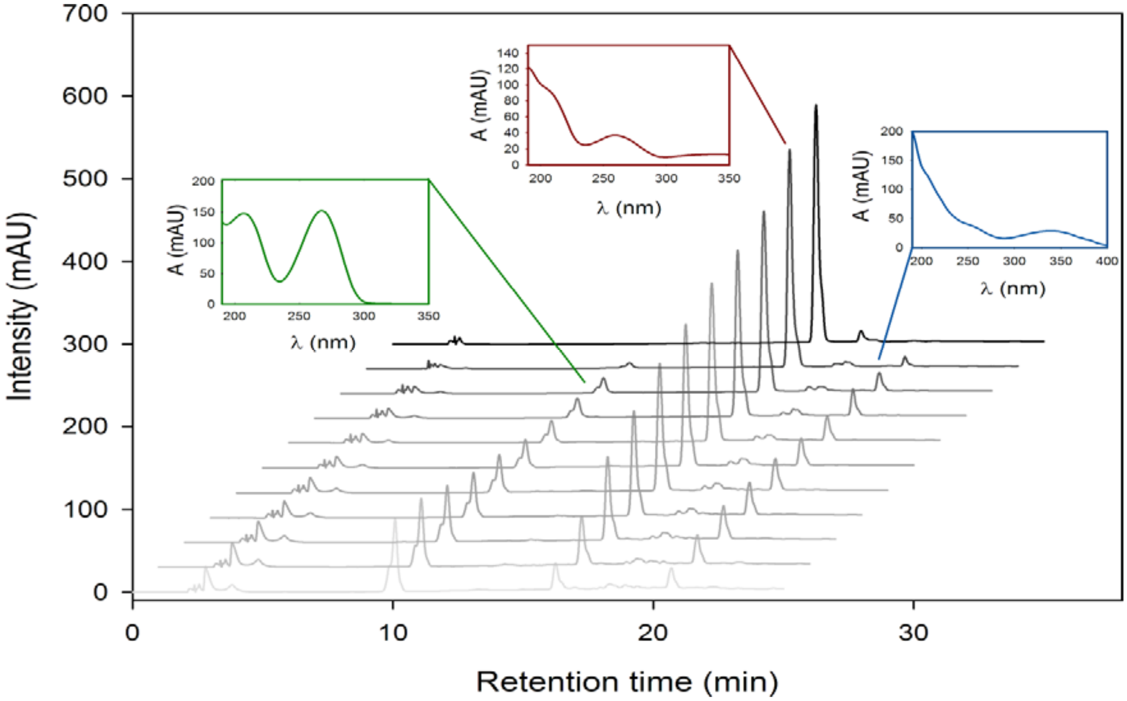

Figure S7. Overlay chromatogram of original SPh-NPPOC sample (back) and increasing exposure degree (front) showing retention time and UV trace of SPh-NPPOC (16.25 min) and exposure products thymidine (10.02 min) and SPh-styrene (20.67 min).

6.d Data analysis and results

The HPLC data was plotted as measured concentration of the starting compound at exposure time t divided by the initial concentration of an unexposed sample aliquot: $c(t)/c(0)$. The absorbance of same exposed samples was used to measure total solution absorbance at 365nm, $A(t)$. The absorbance data was well fitted using a logistics function of the form $A(t)$ = y_0+a/(1+exp(-(t-t_0)/b)). The absorbance data and the fit were then transformed to the photokinetic factor, $(1-10^{-A(t)})/A(t)$. The plots of the $c(t)/c(0)$ and photokinetic factor data and fits are shown in **Figure S8** (green squares and lines). In order to determine the quantum yield of photolysis, the photokinetic factor fit was integrated numerically from 0 to t leaving φ as the single fitting parameter in an equation of the form:

(Eqn. S4)
$$\frac{c(t)}{c(0)} = e^{-k\varphi f(t)}$$

Where *k* is the product of known experimental constants and the molar extinction coefficient and *f*(*t*) are the values corresponding to the numerical integration of the photokinetic factor. **Figure S8** shows the fits of the c(t)/c(0) data using this equation. For NPPOC, Bz-NPPOC and SPh-NPPOC, the values obtained for φ are 0.40, 0.84 and 0.68, respectively.

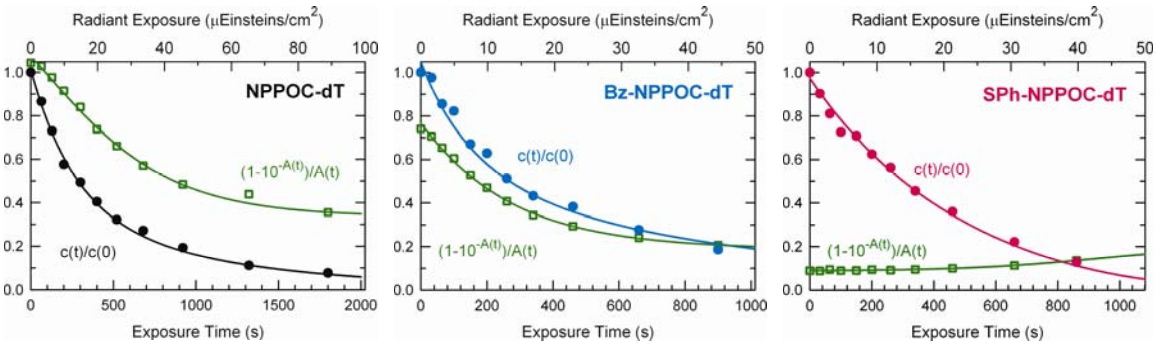

Figure S8. Photolysis kinetics of the NPPOC, Bz-NPPOC and SPh-NPPOC protected thymidines upon irradiation with 365nm light. Irradiance was 16.2mW/cm^2 for NPPOC and Bz-NPPOC and 15.0mW/cm^2 for SPh-NPPOC. c(t)/c(0) data were fitted according to Eqn. S3 and numerical integration of the $(1-10^{-A(t)})/A(t)$ curve.

6.e References

D. Wöll, J. Smirnova, W. Pfleiderer, U. E. Steiner, *Highly Efficient Photolabile Protecting Groups with Intramolecular Energy Transfer*, Angewandte Chemie International Edition, **2006**, 45, 2975.

D. Wöll, J. Smirnova, M. Galetskaya, T. Prykota, J. Bühler, K.-P. Stengele, W. Pfleiderer, U. E. Steiner, *Intramolecular Sensitization of Photocleavage of the Photolabile 2-(2-Nitrophenyl)propoxycarbonyl*

(NPPOC) Protecting Group: Photoproducts and Photokinetics of the Release of Nucleosides, Chemistry – A European Journal **2008**, 14, 6490.

H. Mauser and G. Gauglitz, *Photokinetics: Theoretical Fundamentals and Applications*, in Chemical Kinetics, volume 36, R. G. Compton and G. Hancock, editors, **1998**, Elsevier.
